# Fillet Yield and Length-Weight Relationship of Five Fish Species From Lower Benue River, Makurdi, Nigeria

**DOI:** 10.21315/tlsr2021.32.1.10

**Published:** 2021-03-31

**Authors:** Okomoda Victor Tosin, Solomon Shola Gabriel, Songbe S. Wukatda, Ikape Simon, Ikhwanuddin Mhd, Abol-Munafi Ambok Bolong

**Affiliations:** 1 Department of Fisheries and Aquaculture, College of Forestry and Fisheries, Federal University of Agriculture Makurdi, P.M.B. 2373 Makurdi, Nigeria; 2 Institute of Tropical Aquaculture and Fisheries Research (AQUATROP), Universiti Malaysia Terengganu, 21030 Kuala Nerus, Terengganu, Malaysia; 3 Agricultural Department, National Biotechnology Development Agency (NABDA), Abuja, Nigeria; 4 Department of Agricultural Extension and Management, Federal College of Forestry, Jos. Plateau, Nigeria; 5 Faculty of Food Science and Fisheries, Universiti Malaysia Terengganu, 21030 Kuala Nerus, Terengganu, Malaysia

**Keywords:** Benue River, Carcass Yield, Frame Weight, Length-Weight Relationship

## Abstract

The body characteristics and yield indices of *Clarias gariepinus, Bagrus bajad*, *Synodontis nigrita, Labeo senegalensis* and *Mormyrus rume* from lower Benue River in Nigeria were determined in this study using 60 samples each for the fish species. Length, weight and fillet correlations were also determined during the study. Results obtained showed that *M. rume, L. senegalensis* and *C. gariepinus* had the highest percentage of edible parts (≥ 55%) compared to the other species (≤ 39%). Concerning correlations of the fillet with the morphological variables, results obtained suggest that fillet yield is independent of fish size (except for *C. gariepinus* which was positively correlated). Also, only samples of *L. senegalensis* showed isometric growth pattern; the other fish species had either positive (*C. gariepinus* and *B. bajad*) or negative (*S. nigrita* and *M. rume*) allometric growth. While the difference in fillet yield and body characteristics was attributed to the structural anatomy and other biological dynamics of the fishes, this study could not establish a connection between fillet yield and the length-weight relationship. It was concluded that *M. rume, L. senegalensis* and *C. gariepinus* would be better export products because of their higher fillet yields.

**Highlights**

- Fillet percentage was higher in Mormyrus rume *Labeo senegalensis* and *Clarias gariepinus* (≥ 55%).
- Fillet characters was not significantly correlated with morphological variables for most species studied.
- Isometric growth was observed only in *L. senegalensis*.

## INTRODUCTION

The fisheries industry is dynamic and the changes experienced are geared towards meeting consumer’s diverse needs and preferences. The changing consumers’ taste and advances in technology have made value addition imperative for increased profit (Morris 2013). The high consumption of processed and frozen food is predicated on consumer’s preference for foods requiring less time to prepare, giving them more convenience (Yasemi *et al.* 2011; Costa *et al.* 2014). Hence, the economic importance of marketing fish as packaged fillets has grown exponentially (Saldanha *et al.* 2008). Fillet and carcass yields are very important variables for both the fish processing industry and fish farmers as they can be used to assess the economic value of different fish species (Macedo-Viegas & Souza 2004). Fillet yield is the edible portion of the fish body and therefore excludes the frame, fins, viscera organs and scales (Solomon & Akogu 2005). This, therefore, reduces the bulky transportation of fresh fish products from point of production to the retail shop and saves the consumers the drudgery of cleaning/processing the raw fish before cooking. Sales of fish fillet may not be common in Africa and many developing countries of the world due to infrastructure decadence; however, Food and Agriculture Organization (FAO) (2014) had stated that it is a viable means of introducing tropical fish products into the international markets. Consequently, fish farmers in Sub-Saharan African need to be aware, enlightened and encouraged to produce commercially viable fish fillets to maximise profit.

Many of the fish families in the Benue River (Nigeria) are not commercially relevance. However, despite the high exploitation of family Clarridae, Bagridae, Mochokidae, Cyprinidae and Mormyridae, there is little or no information about their processing traits (fillet yield). Generally, freshwater fish species in Nigeria have been poorly reported with only a few studies carried out in the last few decades (Eyo 1991; Solomon & Akogu 2005; Adeyemo 2013; Ikape & Solomon 2018). Many of these studies, did not report information on the correlation of the different fillet variables with body characteristics. Such studies may be useful for developing models to predict carcass fillet yield of live fish without necessarily killing the fish. This has been demonstrated in earlier studies where fish body characteristics were used as predictors of fillet yield (Rutten *et al.* 2004; Kause *et al.* 2007; Nguyen *et al.* 2010; Thodesen *et al.* 2012). Data collected from fillet yield studies, can also predict the non-edible part and used for the calculation of length-weight relationships (Ikape & Solomon 2018). While the former provides an index to predict the profitability of fish produced, the latter dictates the well-being of the fish during rearing. This study is therefore designed to determine the body characteristics, fillet yield indices (including non-edible parts), the correlation between these variables, and the length-weight relations for some selected freshwater fish species from lower Benue River.

## MATERIALS AND METHODS

**Figure 1:**
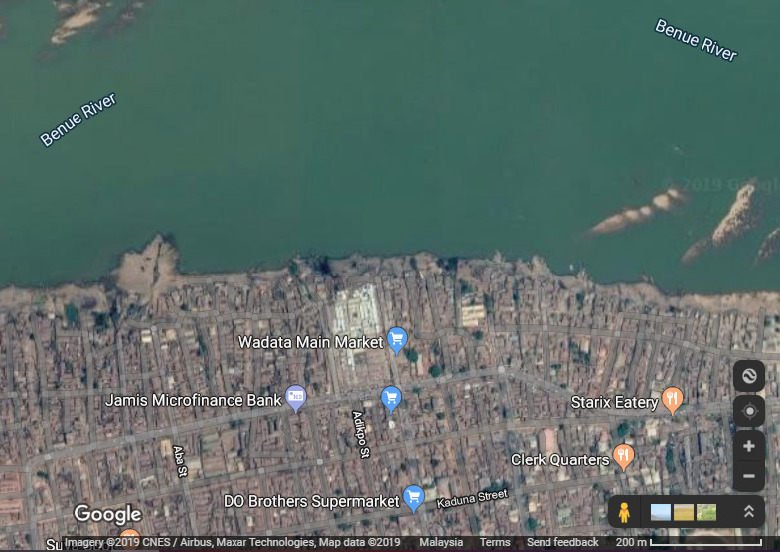
Map of Benue State showing the study area. (*Source*: https://www.google.com.ng/maps)

This study was carried out in the lower Benue River at Makurdi, the capital of Benue State, Nigeria (located at longitude 7°44'43.10''N and latitude 8°30'44.99''E). Benue River is the major tributary of the Niger River and it is approximately 1,400 km long and is almost entirely navigable during the summer months. The fish samples for this study were collected from fishermen operating along the lower Benue River close to the public market place (Fig. 1). Sixty fish samples of *Clarias gariepinus* (Clarridae), *Bagrus bajad* (Bagridae), *Synodontis nigrita* (Mochokidae), *Labeo senegalensis* (Cyprinidae) and *Mormyrus rume* (Mormyridae) were collected (for each species) over six months. The selection of the fish species for each family was based on the commonality and abundance of catches (Solomon *et al.* 2012), market value (high price), and consumer’s preference for these fishes around the Makurdi environ. The collected samples were washed with tap water (to remove any adhering material) and then placed on ice in insulated boxes for preservation. The samples were thereafter transferred to the nutrition laboratory of the Department of Fisheries and Aquaculture, Federal University of Agriculture, Makurdi.

At the laboratory, the biometric variables were recorded. The total length was obtained using a measuring rule (cm) while body weights of the fishes were gotten using an electronic weighing balance (grams). Filleting was done by four skilled fish processors. The procedure for filleting was according to the report of Yenmak *et al.* (2018) following the few modifications made by Okomoda *et al*. (2020). In brief, the scales (i.e., for the *L. senegalensis* with scales) and fins were removed manually from the fish using a sharp knife. The fish were then cut from the top of the head, down the side behind the pectoral fins and along the side of the dorsal fins. The fillet was then removed by cutting along the vertebrae from head to tail. The viscera organs, heads and skeletons (frames) were then separated from fillet left on the cut fish. The various parts were then weighed separately using a sensitive weighing balance and recorded. The percentages of each dressed part (i.e., fillets, head, gut and frame) relative to the weight of whole fish were calculated as follows:

$$\%\;\text{dressed part}=(\frac{\text{Weight of dressed part}}{\text{Total weight}})\times 100$$

Data from each fish species were analysed using Minitab 14. Firstly, descriptive statistics (i.e., means and standard errors) were done for the different variables (fillets, heads, gut and frame) across the species studied. Thereafter, they were subjected to one-way ANOVA. Where significant differences occurred, the means of the fillets, heads, gut and frame were separate across the species using Fisher’s LSD (*P* ≤ 0.05). Pearson’s correlations of the length/weight of the fish and filleting variables were done using the Minitab software. The length-weight relationship of the different fish species was calculated using the equation given by Le Cren (1951) and Ricker (1973) as follows:

LogW=a+b log L

where L = total length in centimetres, W = weight in grams, a = intercept and b = slope.

The hypothesis tested in this study was that the weight of fillets, heads, gut and frame differed among the different species of fish studied.

## RESULTS AND DISCUSSION

The findings of this study show differences in fillet weight (53 g–233 g) and percentages (34%–65%) for the species studied (Tables 1 and 2). Neto *et al.* (2012) had earlier reported 32% fillet for Pacu (*Colossoma bidens*) and Tambaqui (*Colossoma macropomum*). Fillet yield of farmed and wild Nile Tilapia was also ascertained to be between 32% and 37% as reported by Sulieman and James (2011). While in the case of Thailand tilapia (*Oreochromis* spp.) average filleting yield was 31%, ranging between 28.9% and 33.6% (Pinheiro *et al.* 2006). Similarly, different strains of Nile tilapia showed between 31%–38% fillet percentages in the study reported by Rutten *et al.* (2004) and Peterman and Phelps (2012). The fillet yield percentages in our study are also larger than those reported for palmetto bass (33.2%), Jundiá *Rhamdia quelen* (29.5%) and sunshine bass (33.6%–34.9%) by Swann *et al.* (1994), Carneiro *et al.* (2004), and Rudacille and Kohler (2000), respectively. Channel catfish *Ictalurus punctatus* has been reported to have average fillet yields of 30.9% (Clement & Lovell 1994), carp with 53% fillet (FAO 1985), and Coho salmon *Oncorhynchus kisutch* 58% (Neira *et al.* 2004). In other species, fillet percentages varied from 35% in Asian catfish *Pangasianodon hypophthalmus* (Sang *et al.* 2009) and Gilthead Seabream *Sparus auratus* (Navarro *et al.* 2009) to 41% in Common Carp (Kocour *et al.* 2007; Bauer & Schlott 2009) and Channel Catfish (Argue *et al.* 2003). Fillet yields were higher in the studies by Kause *et al.* (2002) for Rainbow trout *O. mykiss* (64%), Powell *et al.* (2008) for Atlantic salmon *Salmo salar* (69%), FAO (1985) for trout (70%) and Oksuz (2010) for cultured Bluefin tuna, *Thunnus thynnus* (73%).

By implication, it would require approximately 15 tonnes of the *M. rume* or *L. senegalensis* to produce 10 tonnes of fillets for consumption/sales, while twice that amount (about 30 tonnes) would have to be caught/produced for *S. nigrita* or *B. bajad* to obtain the same 10 tonnes of the fillet. This, therefore, suggests that *M. rume, L. senegalensis* and *C. gariepinus* may be a better export product in terms of fillet yield compared to *S. nigrita* or *B. bajad*. Generally, the edible portion of fish depends on several factors such as species, sex and size, the structural anatomy of the fish and farming conditions (Alinezhad 2004; González *et al.* 2017). Of all the factors mentioned above, differences in species as it relates to structural anatomy are most likely the culprit of the observable differences reported in the current study. This is because; the highest filleting yields were recorded in flatfishes (i.e., *M. rume* and *L. senegalensis*) as compared to the deep-bodied fishes. In the same light, the lower frame waste of sunshine bass compared to palmetto bass had been explained using the differences in the fish body shape or conformational differences (Mcentire *et al.* 2015). Similarly, Bosworth *et al.* (1998) had earlier justify the significant differences in fillet weight of Asian sea bass and hybrid striped bass using the shape of the anadromous Perciformes species which is slightly compressed and streamlined. Eyo (1991), Ali e*t al.* (1992) and Galvão *et al.* (2010) had earlier opined that species with large heads and skeleton relative to their musculature gave lower filleting yield than those with smaller heads and skeletons. However, Yenmak *et al.* (2018) suggested that the relationship between fish size and fillet yield is positive, meaning, larger fishes provided higher fillet yield percentages than smaller fish size. From the findings of this study, it is clear that this assumption does not hold when different species are considered.

Among the fish examined, *B. bajad* and *S. nigrita* had the highest percentages of inedible parts (≥ 60%, as seen in Tables 1 and 2), followed by *C. gariepinus* and the least observed for *M. rume* and *L. senegalensis* (≤ 37%). In many countries, these inedible parts are either discarded or used to feed carnivorous fishes (especially the visceral organs) or as by-products used as a prime matter for tanning and preparation of animal feeds (Ikape & Solomon 2018). Thus, the commercialisation of these inedible parts can serve as an alternative income source for fisheries industries (Souza *et al.* 2015). It is also noteworthy to mention that discarded inedible fillets are sometimes used as diet by low-in-come people (Eyo 2001). This is because of the significant mass of flesh attached to the bones of discarded frames which could augment the nutrition needed by the poor (Eyo 2001).

Concerning phenotypic correlations, only in *C. gariepinus* were the fillet characteristics having a high positive and significant correlation with the biometric variables (Table 3). Although high positive correlations exist in some of the other species studied, many of the correlations were insignificant. This simply means catching or growing fish to a larger size is not an efficient way to increase fillet weight in these other fishes. This finding is in line with the reports of Bauer and Schlott (2009) and Yasemi *et al.* (2011) who indicated that fillet yield is independent of fish size. Bosworth *et al.* (1998) and Cibert *et al.* (1999) had also concluded that correlation between body weight and fillet weight is generally high, but on the contrary, such correlation between body weight and fillet yield is generally low. Similarly, Rutten *et al.* (2004) experimented on Nile tilapia and observed weak/insignificant relationships between body measurements and fillet yield. This is in contrast to many studies reported by Bosworth *et al.* (1998) and Cibert *et al.* (1999).

Length-weight relationships are indications of the fatness and general well-being of fishes in the different ecosystems (Ikape & Solomon 2018; Okomoda *et al.* 2018). It is well established that length-weight relationships vary even within fishes of the same species as it is affected by sexes, seasons, growth phases, stomach contents, gonadal development, health, etc. (Leunda *et al.* 2006; Gaspar *et al.* 2012). As a result, the reported variation in the current study was not surprising. The findings of this study showed that only *L. senegalensis* had an isometric growth pattern (Table 4). Hence, this implies that its weight increases proportionate to its length (Olufeagba *et al.* 2016). According to Venu and Kurup (2003), this is the “ideal fish” situation, i.e., maintenance of dimensional equality. *C. gariepinus* and *B. bajad*, on the other hand, had positive allometric growth, while negative allometric was observed in *S. nigrita* and *M. rume*. According to Riedel *et al.* (2007), a negative allometric growth implies that the fish is becoming tinnier as it disproportionately increases in length to its weight; hence, the fishes become slender. The values of intercepts “a” and slope “b” recorded for the five fish species in this study were within the range previously reported in other studies (Pervin & Mortuza 2008; Okomoda *et al.* 2018). It is important to state that the differences in the observed length-weight relationship parameter as dictated by the morphological differences of the fish may not necessarily mean that some species are better than the others are. Also, there seems to be no definite trend to portray the connection between the fillet yield and the length-weight relationship parameters. This is because *M. rume* which is one of the fish species with the highest fillet yield in this study had a negative isometric growth pattern similar to *S. nigrita* with the least fillet yield. A detailed study of this observation in a larger number of fish families/species may help justify or debunk this hypothesis.

## CONCLUSION

In conclusion, the differences in fillet yield and body characteristics of the fishes in this study are simply a reflection of structural anatomic differences of the fishes. This has implications for commercial sales of the fishes studied. Judging from the study, we recommend the use of *M. rume, L. senegalensis* and *C. gariepinus* if the exportation of the fish was considered. This is because they can favourably compete with other popular exported fish in terms of fillet yield compared to *S. nigrita* or *B. bajad*. However, this will also be predicated upon the nutritional content of the fish and consumer’s preference, hence the need for more research.

## Figures and Tables

**Table 1:** Mean dressed part of five fish species from lower Benue River.

Species	Whole body weight (g)	Total length (cm)	Gut weight (g)	Head weight (g)	Frame weight (bone) (g)	Edible fillet weight (g)
*C. gariepinus*	424.00 ± 62.2^a^	37.86 ± 1.95^a^	24.22 ± 4.76^a^	124.00 ± 16.2^a^	32.7 ± 4.47^a^	233.0 ± 28.9^a^
*B. bajad*	235.00 ± 57.1^b^	29.73 ± 0.91^b^	6.92 ± 0.78^d^	52.52 ± 4.70^b^	8.26 ± 0.62^b^	91.83 ± 8.95^c^
*S. nigrita*	152.00 ± 14.1^c^	23.28 ± 0.58^c^	16.42 ± 3.58^b^	46.45 ± 3.29^c^	8.27 ± 0.76^b^	53.17 ± 4.70^c^
*L. senegalensis*	160.30 ± 18.9^c^	25.35 ± 0.82^c^	10.66 ± 1.76^c^	23.01 ± 2.43^d^	11.49 ± 1.43^b^	101.0 ± 12.1^b^
*M. rume*	83.26 ± 9.34^d^	24.80 ± 0.81^c^	3.44 ± 0.41^e^	20.26 ± 1.88^d^	6.60 ± 0.74^b^	77.96 ± 25.0^c^

*Notes*: Mean in the same column with different superscript differ significantly (*P* < 0.05). The numbers in each cell are means ± standard error.

**Table 2:** Percentage fillet composition of five fish species from lower Benue River.

Species	% gut weight	% head weight	% frame weight (bone)	% edible fillet weight	% non-edible fillet
*C. gariepinus*	5.71 ± 0.05^bc^	29.18 ± 0.21^a^	7.72 ± 0.15^a^	55.01 ± 1.05^b^	44.95 ± 0.13^c^
*B. bajad*	2.95 ± 0.02^d^	22.36 ± 0.11^b^	3.52 ± 0.05^c^	39.09 ± 0.15^c^	60.90 ± 0.40^b^
*S. nigrita*	10.80 ± 0.10^a^	30.54 ± 0.25^a^	5.44 ± 0.09^b^	34.96 ± 0.23^d^	65.05 ± 0.12^a^
*L. senegalensis*	6.65 ± 0.03^b^	14.34 ± 0.95^c^	7.17 ± 0.03^a^	63.20 ± 0.41^a^	36.65 ± 0.49^d^
*M. rume*	4.13 ± 0.01^c^	20.33 ± 0.10^b^	7.93 ± 0.11^a^	65.03 ± 1.02^a^	34.99 ± 0.21^d^

*Notes*: Mean in the same column with different superscript differ significantly (*P* < 0.05). The numbers in each cell are means ± standard error.

**Table 3:** Correlation of some fillet and body variables of five fish species from lower Benue River.

Fish species	TBW/HW	TBW/FW	TBW/BFW	TBW/GW	TBW/TBL	TBL/HL	TBL/GW
*C. gariepinus*	0.968**	0.964**	0.951**	0.835**	0.912**	0.927**	0.766**
*B. bajad*	0.382	0.389	0.479*	0.201	0.572**	0.937**	0.697**
*S. nigrita*	0.576**	0.343	0.319	0.416*	0.608**	0.45*	0.229
*L. senegalensis*	0.335	0.243	0.471*	0.182	0.943**	0.583**	0.223
*M. rume*	0.821**	0.115	0.746**	0.277	0.805**	0.693**	0.484*

*Notes*: TBW=Total body weight; TBL = Total body length; HW = Head weight; FW = Fillet weight; BFW = Body frame weight; GW = Gut weight; HL = Head length.

** Correlation is significant at 0.001;

* Correlation is significant at 0.01.

The numbers in each cell are means ± standard error.

**Table 4:** Length-weight relationship of five fish species from lower Benue River.

Parameter	a	b	*r*^2^
*C. gariepinus*	−2.601	3.239	0.601
*B. bajad*	−3.786	4.096	0.609
*S. nigrita*	−1.283	2.511	0.593
*L. senegalensis*	−2.071	3.024	0.906
*M. rume*	−1.014	2.071	0.832

*Note*: a = Intersect; b = Slope; *r*^2^ = Regression coefficient probability. The numbers in each cell are values of linear regression analysis.
